# The relationship between depressive symptoms and sleep quality in medical staff after their infection with COVID-19

**DOI:** 10.3389/fpsyt.2023.1269402

**Published:** 2023-11-30

**Authors:** Amirhossein Memarian, Parvin Mangolian Shahrbabaki, Mohammad Ali Zakeri, Mehdi Ahmadinejad

**Affiliations:** ^1^Nursing Research Center, Kerman University of Medical Sciences, Kerman, Iran; ^2^Pistachio Safety Research Center, Rafsanjan University of Medical Sciences, Rafsanjan, Iran; ^3^Clinical Research Development Unit, Ali-Ibn Abi-Talib Hospital, Rafsanjan University of Medical Sciences, Rafsanjan, Iran; ^4^Department of Anesthesiology, School of Medicine, Shahid Bahonar Hospital, Kerman University of Medical Sciences, Kerman, Iran

**Keywords:** COVID-19, depression, sleep quality, sleep disorders, medical staff

## Abstract

**Background:**

Healthcare workers (HCWs) play a crucial role in managing infectious diseases like COVID-19. However, the demanding working conditions during the pandemic have led to an increased risk of depression and sleep disorders among these dedicated professionals. Therefore, this study aimed to examine the relationship between depressive symptoms and sleep quality in medical staff who had contracted COVID-19.

**Methods:**

This descriptive study involved a sample of 203 HCWs who had contracted COVID-19. These HCWs were employed at a hospital affiliated with Kerman University of Medical Sciences in 2020. The data for this study were collected using a demographic information form, the Pittsburgh Sleep Quality Index (PSQI), and the Beck Depression Inventory (BDI). Statistical analysis was conducted using SPSS22, with a significance level set at less than 0.05.

**Results:**

The study found that the mean score for depressive symptoms among the participants was 11.67 ± 2.68, while the mean score for sleep quality was 5.47 ± 3.02. It was observed that 18.2% of the participants experienced moderate depression, 10.3% had severe depression, and 59.6% had poor sleep quality. Furthermore, a significant and positive correlation was identified between sleep quality and depression (*r* = 0.54; *p* = 0.001). Multiple regression models indicated that the harmful pattern and sleep quality together could predict 34% of the variance in depression. Additionally, the use of sedatives and depression were found to predict 33% of the variance in sleep quality.

**Conclusion:**

The findings of our study indicated a high prevalence of depressive symptoms and insomnia among medical staff who had contracted COVID-19. These results provide valuable insights for health managers, highlighting the need for implementing interventions in epidemic environments to reduce the vulnerability of HCWs.

## Introduction

Coronavirus disease 19 (COVID-19) is known to cause a wide range of symptoms, ranging from mild cold-like symptoms to acute respiratory syndromes, and in some cases, it can lead to death due to pneumonia and respiratory complications (1). Similar to other RNA viruses, coronaviruses exhibit significant genetic diversity and have a high recombination rate, allowing them to rapidly spread among humans and animals worldwide (2). The outbreak of COVID-19, a group of acute respiratory diseases with an unknown cause, originated in Wuhan, Hubei Province, China in December 2019 (3, 4). The World Health Organization (WHO) declared COVID-19 as a pandemic on March 11, as the virus continued to spread rapidly across the world (5). On January 20, China confirmed cases of COVID-19 transmission among HCWs in Wuhan (6). Apart from the physical impact, the disease has also had a significant impact on the mental health of individuals (7). Studies have shown that when HCWs face an excessive workload coupled with the risk of contracting the disease, the resulting double stress can have detrimental effects on their health and organizational performance (8). High levels of stress can impair cognitive function, concentration levels, and clinical practice (9).

Individuals who have had COVID-19 often report a wide range of symptoms that persist for months after the acute infection, including psychiatric symptoms such as anxiety, depression (10, 11), suicidal thoughts, and mood changes (12). It is important to note that late neurological manifestations following SARS-CoV-2 infection are just as likely as persistent neurological symptoms (13). Therefore, it is crucial to closely monitor and observe late neuropsychiatric manifestations in order to plan preventive interventions (14).

Stress, anxiety, and depression pose significant challenges for psychologists, psychiatrists, and behavioral scientists worldwide, with depression being the most common mental disorder in the world (15). Individuals with depression experience persistent feelings of sadness and hopelessness. Symptoms of depression include a depressed mood, loss of interest in previously enjoyable activities, changes in weight, decreased cognitive and physical functioning, feelings of fatigue, worthlessness, guilt, thoughts of suicide, and death (16, 17). Depression can significantly affect the quality of life, concentration, sleep quality, and overall functional efficiency, and it has a negative impact on personal and social aspects of life (18, 19). Recent studies indicated that approximately 23% of HCWs experienced depression during the COVID-19 outbreak (20). Iranian studies have also suggested an increase in depression among HCWs (10, 11). According to new theories, the systemic inflammation caused by COVID-19 may contribute to the development of depression in individuals who have recovered from the disease (21). Studies conducted in China have reported that HCWs working during the COVID-19 epidemic are at risk of experiencing negative effects on their physical and mental health, with anxiety and stress having a detrimental impact on sleep quality (22). Many HCWs who have had COVID-19 may experience mental and psychological problems, as well as disturbances in sleep quality (23).

Sleep plays a crucial role in maintaining the quality of life and daily functioning, as it directly affects cognitive function and concentration levels during daily activities (24). Sleep quality is an important clinical but a complex phenomenon that is difficult to define and its measure is subjective. One subjective indicator associated with sleep is wake after sleep onset (WASO) (25), which can lead to nervousness, behavioral changes, physiological disruptions, and a reduced capacity to cope with daily stress. Additionally, it can contribute to self-care disorders (25). Recent studies have indicated that sleep disorders are prevalent among HCWs, with approximately 34.32% experiencing sleep disorders (20). The overall prevalence of sleep disorders in the general population is estimated to be around 30% (26). Numerous studies have highlighted the impact of mental health and sleep on the immune system. Adequate and good-quality sleep can help enhance immunity against viral infections, and there is a close relationship between sleep quality and immune system function (27).

Comparisons between different professional groups have consistently shown that HCWs have the poorest sleep quality, particularly when they work longer shifts (28). Sleep quality has a significant impact on mental performance, and inadequate sleep can result in reduced alertness, impaired attention, and slower cognitive processing (29). Prolonged work hours combined with chronic sleep deprivation can lead to cognitive impairments such as slow thinking, memory loss, weakness, irritability, and even depression and suicidal thoughts. Nurses, in particular, are directly affected by professional stress, which negatively affects their sleep quality (29). It has been observed that higher levels of stress are associated with poorer sleep quality (30). Sleep problems are not only detrimental to individuals’ well-being but also have significant medical and financial costs. Healthcare systems worldwide spend substantial amounts of money on compensating for damages related to sleep problems (31).

During the COVID-19 pandemic, HCWs faced a significant psychological burden, particularly when they themselves became infected and experienced clinical symptoms. It is crucial for the government to provide necessary facilities and support to reduce anxiety, depression, and improve the overall mental health of HCWs (32). Studies have shown that the prevalence of depression and insomnia among HCWs during the COVID-19 outbreak is significantly higher compared to the general population (10). HCWs who have contracted COVID-19 may be more susceptible to various psychological consequences of the outbreak, leading to higher levels of depression and insomnia. However, there is a lack of research addressing mental health outcomes specifically in HCWs who have had COVID-19. Therefore, the present study aimed to investigate the relationship between depressive symptoms and sleep quality in HCWs after their infection with COVID-19.

## Method

### Study design and setting

This cross-sectional, descriptive, and correlational study aimed to explore the association between depressive symptoms and sleep quality among HCWs who had contracted COVID-19. The study was conducted at a hospital affiliated with Kerman University of Medical Sciences, specifically Bahonar Hospital. This hospital was chosen due to its convenient sample accessibility and the cooperation of its personnel. The study included all HCWs who had a history of COVID-19 infection. The questionnaire was administered after a recovery period of 3 to 4 weeks following the active stage of the COVID-19 infection, ensuring that the participants had fully recuperated.

The inclusion criteria for this study consisted of HCWs (physicians and nurses) who had initially shown positive symptoms in their chest CT scan and tested positive for COVID-19 through RT-PCR. These participants had returned to their workplace after recovering from the infection. On the other hand, the exclusion criteria included individuals who were unable to participate in the study due to pre-existing mental health issues, diagnosed with other viral respiratory diseases, reported a history of depression and sleep disorders prior to the disease, and those who had incomplete questionnaire responses. The study employed a census method, and initially, 220 HCWs agreed to participate. However, 5 individuals declined to continue, and 12 participants were excluded due to incomplete questionnaire completion. Therefore, a total of 203 questionnaires were included in the final analysis.

### Measurements

Data were collected using a three-part questionnaire: a demographic information form, the Pittsburgh Sleep Quality Index (PSQI), and the Beck Depression Inventory (BDI).

### Demographic information form

The demographic information included sex, age, marital status, job, education level, history of addiction, use of sedatives, and the diagnostic method of the COVID-19.

### Pittsburgh sleep quality index

The Pittsburgh Sleep Quality Index (PSQI), developed by Buysse et al. (33), is a 19-item questionnaire used to assess sleep quality (34). Each item is scored on a 4-point Likert scale ranging from 0 to 3. The questionnaire consists of seven subscales: (1) subjective sleep quality, (2) sleep latency, (3) sleep duration, (4) habitual sleep efficiency, (5) sleep disturbances, (6) use of sleep medication, and (7) daytime dysfunction.

The scoring for each subscale is as follows:

- Questions 1 and 3: No scoring, but the obtained numbers are used in the calculation of other scales.
- Question 2: Response options include ≤15 min (score 0), 16–30 min (score 1), 31–60 min (score 2), and > 60 min (score 3).
- Question 4: Response options include >7 h (score 0), 6–7 h (score 1), 5–6 h (score 2), and < 5 h (score 3).
- For the remaining questions: Response options include no problem at all (score 0), once a week (score 1), twice a week (score 2), and three or more times a week (score 3).

Individual scores for each item range from 0 (indicating no problem) to 3 (indicating a significant problem). The total score for the questionnaire is obtained by summing the scores of the seven subscales, resulting in a total score between 0 and 21. A total score higher than 5 indicates poor sleep quality (34).

The authors of the PSQI have reported a favorable level of validity for this questionnaire, with a sensitivity of 68% and specificity of 86.5%. The reliability of the PSQI was assessed using the bisection method and the Cronbach’s alpha coefficient, resulting in a coefficient of 0.79 (24). In a study conducted by Izadi et al. (35), the validity and reliability of the PSQI were investigated. The retest reliability of the instrument was assessed, yielding a Cronbach’s alpha coefficient of 0.80 (35).

### Beck depression inventory

The Beck Depression Inventory (BDI) was developed by Aaron T. Beck to assess the presence and severity of depression in patients. It consists of 21 multiple-choice self-report items that measure various symptoms of depression, including hopelessness, irritability, guilt, physical symptoms (such as fatigue, weight loss, and lack of interest in sex), and other related factors.

Each answer on the BDI is scored on a scale value of 0–3. The total score on this questionnaire ranges from 0 to 63. Scores between 0–7 indicate no depression, scores between 8–15 indicate mild depression, scores between 16–25 indicate moderate depression, and scores between 26–63 indicate severe depression.

The BDI has demonstrated good reliability, with a Cronbach’s alpha coefficient of 0.89, indicating high internal consistency. Additionally, its concurrent correlation with the BDI-21 (a shorter version of the BDI) was found to be 0.76, suggesting good concurrent validity. Overall, the BDI has shown favorable reliability and validity for assessing depression symptoms (36).

### Data collection

After obtaining the required permissions, the researcher conducted the sampling process at Bahonar Hospital in Kerman. Data collection took place during various shifts to ensure a diverse sample. The researcher coordinated with physicians and nurses who had previously been infected with COVID-19 and had returned to work after recovery. They explained the study’s objectives and methodology to the participants. The questionnaires were completed when the physicians and nurses were willing and had sufficient time, which typically took around 15 to 20 min. Data collection was carried out through face-to-face interviews. The sampling process took place between April 2021 and June 2022.

### Statistical analysis

For data analysis, the researchers utilized SPSS22. Descriptive statistics, including frequency, percentage, mean, and standard deviation, were employed to describe the background characteristics of the research participants. To investigate the relationship between the PSQI and BDI scores and the participants’ background characteristics, various statistical tests were used. These included independent t-test, Mann–Whitney U test, Kruskal-Wallis H test, and analysis of variance (ANOVA) test.

Furthermore, multiple regression analysis was conducted to identify predictors of sleep quality. Pearson’s correlation coefficient was used to examine the relationship between depression and sleep quality. A significance level of 0.05 was considered to determine statistical significance in all analyses.

### Ethical issue

The present study was conducted in compliance with ethical standards and received approval from the research ethics committee of Kerman University of Medical Sciences, with the code of ethics being IR.KMU.AH.REC.1400.116. All experimental protocols involving human participants adhered to national and institutional guidelines, as outlined in the manuscript. Before conducting the research, the researcher obtained written informed consent from all participants. They were provided with a clear explanation of the study objectives, assured of the confidentiality of their information, and informed of their right to withdraw from the study at any time.

## Results

The study included a total of 203 HCWs employed in hospitals affiliated with Kerman University of Medical Sciences. Among them, 117 (57.6%) were women and 86 (42.4%) were men. The participants had a mean age of 31.18 ± 10.96 years and a mean work experience of 6.28 ± 3.22 years. The majority of participants were single (61.1%) and physicians (77.3%). A small percentage of participants (2.5%) reported addiction, while 5.4% reported using sedatives. Most participants’ COVID-19 diagnoses were confirmed through PCR tests (54.2%) (Table 1).

**Table 1:** Demographic variables of the participants and their relationship with depression and sleep quality (*n* = 203).

Variables	Mean (SD)	Depression		Sleep quality	
		Pearson correlation coefficient	*p* value	Pearson correlation coefficient	*p* value
Age (yr.)	31.18 (10.95)	−0.19	0.007*	0.02	0.20
Work experience (yr.)	6.28 (3.22)	−0.15	0.03*	−0.01	0.07
	***N* (%)**	**Mean ± SD**	***p* value**	**Mean ± SD**	***p* value**
Gender					
Male	86 (42.4)	11.13 ± 1.27	0.54	5.12 ± 3.23	0.16
Female	117 (57.6)	12.05 ± 0.90		5.73 ± 2.85	
Marital status					
Single	124 (61.1)	12.83 ± 1.03	0.07	5.63 ± 3.22	0.61
Married	75 (36.9)	10.17 ± 1.05		5.25 ± 2.75	
Widowed	4 (2.0)	3.75 ± 1.31		4.75 ± 0.95	
Educational level					
Bachelor	38 (18.7)	11.65 ± 1.47	0.46	5.31 ± 2.48	0.83
Masters and PhD	8 (3.9)	7.12 ± 1.32		5.00 ± 1.51	
Physician and specialist	157 (77.3)	11.90 ± 0.89		5.54 ± 3.20	
Job status					
Medicine	157 (77.3)	11.90 ± 0.89	0.56	5.54 ± 3.20	0.58
Nursing	46 (22.7)	10.86 ± 1.25		5.26 ± 2.33	
Participants’ harmful pattern					
Yes	5 (2.5)	24.00 ± 5.44	0.009*	6.20 ± 4.32	0.59
No	198 (97.5)	11.35 ± 0.74		5.45 ± 3.00	
Taking sedative medication					
Yes	11 (5.4)	11.54 ± 2.79	0.96	7.18 ± 2.96	0.05
No	192 (94.7)	11.67 ± 0.77		5.38 ± 3.01	
How to diagnose COVID-19					
Medicine	85 (41.9)	11.10 ± 1.15		5.28 ± 2.83	
PCR	110 (54.2)	12.25 ± 1.02	0.65	5.63 ± 3.17	0.71
CT – SCAN	8 (3.9)	9.62 ± 4.10		5.37 ± 3.15	

Data were presented as number (%).*Significance level less than 0.05.

The mean score for depressive symptoms among the participants was 11.67 ± 2.68, indicating mild depression (15-8). Among the participants, 41.9% were classified as normal, 29.6% had mild depression, 18.2% had moderate depression, and 10.3% had severe depression. The mean sleep quality score was 5.47 ± 3.02, indicating significant sleep problems. Furthermore, 59.6% of participants had poor sleep quality, while 40.4% had normal sleep quality.

Table 1 demonstrated a significant positive relationship between depression, age, and work experience, indicating that higher age and work experience were associated with higher levels of depression. Additionally, a significant relationship was found between harmful pattern and depressive symptoms. However, no significant associations were observed between sleep quality, gender, marital status, education level, occupation, addiction, use of sedatives, and diagnostic method. There was also no relationship between sleep quality, age, and work experience.

The study revealed a significant positive relationship between sleep quality and depressive symptoms (*r* = 0.54, *p* = 0.001), indicating that higher levels of depressive symptoms were associated with poorer sleep quality. All dimensions of sleep quality, except sleep efficiency, showed significant positive associations with depressive symptoms (*p* < 0.05).

In our study, multiple regression models were employed to assess the predictive value of demographic variables on depressive symptoms. The results indicated that only the harmful pattern and sleep quality variables were significant predictors, accounting for 34% of the variance in depressive symptoms (Table 2). Similarly, multiple regression models were utilized to examine the predictive value of demographic variables on sleep quality. As shown in Table 3, the use of sedatives and depressive symptoms were the only significant predictors, explaining 33% of the variance in sleep quality.

**Table 2:** Multiple regression analysis summary for underlying variables of depression among the participants (*n* = 203).

Variable		Unstandardized coefficients		Standardized coefficients	*t*	*p*	95% CI lower	95% CI upper	*R* ^2^
		β	Std Error	β					
Depression	Age	−0.06	0.15	−0.06	−0.39	0.69	−0.35	0.23	%34
	Gender	0.67	1.37	0.03	0.49	0.62	−2.03	3.38	
	Marital status	−1.85	1.3	−0.15	−1.42	0.15	−4.43	0.71	
	Educational level	−4.12	3.47	−0.3	−1.18	0.23	−10.98	2.73	
	Job status	−7.93	6.56	−0.31	−1.21	0.22	−20.88	5	
	Work experience	0.05	0.17	0.04	0.29	0.76	−0.29	0.4	
	Addiction	−10.58	4.09	−0.15	−2.58	**0.01**	−18.66	−2.50	
	Taking sedative medication	2.44	2.94	0.05	0.83	0.4	−3.37	8.26	
	How to diagnose COVID-19	0.28	1.15	0.01	0.24	0.8	−1.99	2.55	
	Sleep quality	1.87	0.21	0.52	8.69	**0.001**	1.44	2.29	

Data were presented as multiple regression analysis; CI, confidence intervals for B. Bold value represents the relationship of variables that were statistically significant.

**Table 3:** Multiple regression analysis summary for underlying variables of sleep quality among the participants (*n* = 203).

Variable		Unstandardized coefficients		Standardized coefficients	*t*	*p*	95% CI lower	95% CI upper	*R* ^2^
		β	Std error	β					
Sleep quality	Age	−0.07	0.04	−0.25	−1.69	0.09	−0.15	0.01	%33
	Gender	0.34	0.39	0.05	0.89	0.37	−0.42	1.11	
	Marital status	0.31	0.37	0.06	0.83	0.40	−0.42	1.04	
	Educational level	0.23	0.99	0.06	0.23	0.81	−1.72	2.19	
	Job status	0.19	1.87	0.02	0.10	0.99	−3.49	3.89	
	Work experience	0.04	0.05	0.13	0.89	0.37	0.05	0.14	
	Addiction	1.25	1.18	0.06	1.06	0.28	−1.07	3.58	
	Taking sedative medication	−1.71	0.83	−0.12	−2.06	**0.04**	−3.35	−0.08	
	How to diagnose COVID-19	0.26	0.32	0.05	0.81	0.41	−0.37	0.91	
	Depression	0.15	0.01	0.53	8.69	**0.001**	0.11	0.18	

Data were presented as multiple regression analysis; CI, confidence intervals for B. Bold values represent the relationship of variables that were statistically significant.

## Discussion

This study aimed to investigate the presence of depressive symptoms and sleep quality among HCWs who had contracted COVID-19 and were working in hospitals affiliated with Kerman University of Medical Sciences in 2022. The results of the study revealed that 28.5% of the participants experienced moderate to severe depression. Klaser et al. also found that the overall prevalence of anxiety and depressive symptoms in the general population of the UK was 26.4% compared to before the pandemic. The rate of anxiety and depression was slightly higher in individuals who tested positive for severe acute respiratory syndrome coronavirus 2 (SARS-CoV-2) (30.4%) compared to those who tested negative (26.1%) (37). Wang et al. (38) reported that 35% of HCWs in China exhibited symptoms of depression during the COVID-19 outbreak. In contrast to our study, Behnoush et al. indicated that 42.4% of Iranian nurses with COVID-19 reported experiencing depression (32), which was a higher percentage than observed in our study. Zakari et al. found that 18.40% of the participants experienced depression during the COVID-19 outbreak (39), which was a lower percentage than observed in the present study. Our study specifically focused on HCWs who had contracted COVID-19 and were working under challenging treatment conditions. The COVID-19 epidemic has had a significant impact on the quality of work and safety of healthcare workers, increasing the risk of infection. The direct involvement of HCWs with COVID-19 patients has also had a negative effect on their psychological well-being, potentially contributing to higher levels of depression. Some studies have suggested a significant increase in anxiety, stress, and depression among nurses during the initial wave of the COVID-19 pandemic compared to before the outbreak (40). The variations in study results may be attributed to the use of different assessment scales and cutoff points in each questionnaire, cultural differences, and the specific conditions of the healthcare system in different regions, which can influence the mental state of nurses affected by COVID-19. Based on the findings of this study, a majority of HCWs experienced mild to severe symptoms of depression (approximately 60%), highlighting the importance of early diagnosis and treatment of depression to prevent the development of more complex psychological responses.

The study results showed that 59.6% of the participants experienced poor sleep quality. As there were no studies specifically investigating sleep quality among HCWs with COVID-19, similar studies were used for comparison. Wang et al. conducted a study in China (38) and reported that about 61.6% of HCWs experienced sleep problems during the COVID-19 outbreak, with frontline HCWs having a higher prevalence of sleep disorders compared to non-frontline and non-HCWs. Yılmaz et al. (41) in Turkey supported our results and found that the overall prevalence of poor sleep quality during the COVID-19 outbreak was 56.7%. Among nurses, physicians, and dentists, the prevalence of poor sleep quality was 67.3%, 55.4%, and 42.3%, respectively (41). Huang and Zhao (42) in China also found that HCWs had poorer sleep quality compared to the general population. Similarly, Wang et al. (38) in China reported a higher prevalence of sleep disorders among frontline HCWs compared to non-frontline and non-HCWs during the COVID-19 outbreak. Jahrami et al. (43) in Bahrain demonstrated that 75% of frontline HCWs and 76% of non-frontline HCWs experienced poor sleep quality during the COVID-19 outbreak. Other studies conducted in Iran suggested that 49.7% of nurses experienced insomnia during the COVID-19 outbreak (39), showing a lower percentage of sleep problems in HCWs. However, a survey conducted in the general community in Iran showed that 26.8% experienced sleep disorders during the COVID-19 outbreak (7). These findings highlight the high prevalence of sleep problems among HCWs. Therefore, healthcare managers should prioritize addressing sleep problems and their consequences by implementing interventions to reduce them.

The present study found a significant and direct correlation between depression and sleep quality, which is supported by the existing literature. Pappa et al. examined the impact of the COVID-19 outbreak on the sleep and mood of HCWs (20). Xiao et al. in China investigated the effect of anxiety and depression on sleep disorders among HCWs during the COVID-19 epidemic (22). Deng et al. demonstrated that stress had a negative impact on the sleep quality of Chinese nurses (44). Coping with stressful life events can lead to psychiatric disorders, which can affect the concentration, perception, decision-making abilities, and overall well-being of HCWs (7). Furthermore, HCWs may be more susceptible to psychological distress due to their close contact with patients. The present study revealed a decrease in sleep quality associated with an increase in the level of depression, which aligns with the findings reported in the literature. Early interventions are necessary to improve the mental health of HCWs (32), as they bear a significant psychological burden during the COVID-19 pandemic, particularly when they themselves become infected and experience clinical symptoms. Therefore, it is crucial for the government to provide resources and support to reduce depression and enhance the mental well-being of HCWs.

The study findings revealed that the participants’ dependency pattern and sleep quality accounted for 34% of the variance in depression. Yılmaz et al. (41) also conducted a multivariate regression analysis and found a significant correlation between poor sleep quality, working in hospitals, and high levels of traumatic stress during the COVID-19 pandemic. However, other studies have identified additional variables that contribute to mood disorders such as anxiety and stress. Jahrami et al. (43) in Bahrain demonstrated that female gender and work experience were predictors of stress during the COVID-19 outbreak (43). Huang and Zhao (42) in China found an association between age (less than 35 years), time spent on COVID-19 (≤ 3 h per day), and anxiety disorder (39). The review of literature indicates that sleep problems, particularly insomnia, play a role in the development of mood disorders, suggesting that insomnia can be a predictor of anxiety and depression, and vice versa (45). Effective improvement of sleep disorders may help reduce the incidence of subsequent or concurrent mood disorders, and vice versa (46). These findings highlight the reciprocal relationship between sleep quality and depression, where each can be both a consequence and a predictor of the other.

The study findings indicated that the use of sedatives and depressive symptoms accounted for 33% of the variance in sleep quality. Hyun et al. (47) conducted a study in China that supported our results and found that symptoms of depression and anxiety were predictors of sleep quality, regardless of any previous diagnosis. Yang et al. (48) also found an association between anxiety, depression, and poor sleep quality. However, Jahrami et al. (43) in Bahrain reported that female gender and work experience were predictors of poor sleep quality during the COVID-19 outbreak (43). Wang et al. (38) in China demonstrated that frontline HCWs were more susceptible to sleep disorders during the COVID-19 outbreak. Therefore, it is crucial to implement psychosocial interventions to support HCWs in effectively responding to the challenges posed by COVID-19 and future outbreaks.

### Research limitations

The main strength of our study lies in its focus on frontline HCWs who contracted COVID-19, as they experienced the most challenges and complications during the epidemic. Policymakers can utilize the findings of this study to develop more effective programs aimed at improving the well-being of affected HCWs. However, it is important to note that we encountered difficulties in accessing HCWs during the pandemic, and we made efforts to reach them during different shifts. It is worth mentioning that all participants were employed at Bahonar Hospital, which limits the generalizability of our results to other healthcare institutions in Iran. Therefore, we recommend conducting further studies with larger populations.

In the current study, we employed a self-report questionnaire to assess depression and sleep quality. Additionally, in the case of participants reporting harmful patterns, the type of sedative used was self-reported and not specified. Therefore, caution must be exercised when interpreting these results.

An important aspect highlighted in this study is the need for better planning and increased attention towards HCWs during crises, with a focus on preventing harm to them. As frontline healthcare providers, they play a crucial role in the treatment and maintenance of public health. By prioritizing the well-being of HCWs, the quality of care provided can be significantly enhanced.

## Conclusion

The study findings revealed that HCWs experienced elevated levels of depression and insomnia, indicating a substantial psychological burden during the COVID-19 pandemic. This burden was particularly pronounced when HCWs themselves contracted the virus and experienced clinical symptoms. Consequently, it is imperative for the government to offer resources and administrative support aimed at mitigating depression and enhancing the mental well-being of HCWs.

The current study has contributed novel insights into the challenges confronted by HCWs affected by COVID-19. These findings can aid in quantifying the support requirements of HCWs and implementing targeted interventions to alleviate their vulnerability.

## Clinical practice and medicine

HCWs have been significantly affected by various psychological issues, including depressive symptoms and insomnia, during the Covid-19 pandemic. It is crucial to prioritize the recognition and management of late physical and psychological manifestations in individuals who have recovered from SARS-CoV-2 infection in the post-corona era. This is essential to prevent complications and facilitate the rehabilitation of those who have been infected. HCWs, due to their challenging working conditions, are at a higher risk of experiencing exacerbated late complications, particularly when they themselves contract the virus. Therefore, it is of utmost importance to identify these complications early on and provide rehabilitation support to minimize their impact.

The present study sheds light on the challenges faced by HCWs in the post-coronavirus era and the long-term problems and complications resulting from this disease. The results convey an important message to readers and managers alike. It highlights the causal relationship between depressive symptoms and sleep disorders, where insomnia can contribute to depression and vice versa. If left untreated, depressive symptoms can progressively worsen sleep quality, ultimately leading to a depressed mood and low quality of life. Therefore, it is crucial to implement specialized care programs during crises such as the Covid-19 pandemic, with a focus on improving the quality of sleep and reducing depression among HCWs.

## Data availability statement

The raw data supporting the conclusions of this article will be made available by the authors, without undue reservation.

## Ethics statement

The studies involving humans were approved by Kerman University of Medical Sciences. The studies were conducted in accordance with the local legislation and institutional requirements. The participants provided their written informed consent to participate in this study.

## Author contributions

AM: Conceptualization, Data curation, Investigation, Methodology, Writing – original draft. PS: Conceptualization, Formal analysis, Investigation, Methodology, Supervision, Writing – review & editing, Validation. MZ: Formal analysis, Writing – original draft, Writing – review & editing, Investigation, Methodology. MA: Conceptualization, Investigation, Methodology, Supervision, Validation, Writing – review & editing.
